# CT - derived fractional flow reserve can predict recurrent ischemia in patients with MCA stenosis

**DOI:** 10.3389/fneur.2026.1838968

**Published:** 2026-06-17

**Authors:** Zhuhao Yin, Tongyuan Liu, Changsheng Zhou, Jian Guo, Yuan Wei, Yifei Ma, Fan Zhou, Wusheng Zhu, Xiaoqing Cheng, Long Jiang Zhang

**Affiliations:** 1 Department of Radiology, Jinling Hospital, Nanjing Medical University, Nanjing, Jiangsu, China; 2 Department of Radiology, Jinling Hospital, Affiliated Hospital of Medical School, Nanjing University, Nanjing, Jiangsu, China; 3 Shanghai United Imaging Medical Technology Group Co., Ltd., Shanghai, China; 4 Department of Neurology, Jinling Hospital, Affiliated Hospital of Medical School, Nanjing University, Nanjing, Jiangsu, China

**Keywords:** computational fluid dynamics, CT perfusion, fractional flow reserve, intracranial arterial stenosis, prognosis

## Abstract

**Rationale and objectives:**

CT-derived fractional flow reserve (CT-FFR) has rarely been used to evaluate the hemodynamic changes of cerebral arterial stenosis. We aimed to investigate the role of CT-FFR in predicting recurrent ischemic risk in patients with symptomatic middle cerebral artery (MCA) stenosis.

**Materials and methods:**

124 eligible patients with unilateral MCA stenosis undergoing four-dimensional CTA were retrospectively included. Computational fluid dynamics model was reconstructed from the peak arterial phase CTA to calculate CT-FFR. MTT > 6.5 s was defined as cerebral hypoperfusion. Recurrent ischemic event within 1 year was recorded.

**Results:**

Of 124 patients, 19 had recurrent ischemic event, with lower CT-FFR value than non-recurrent ischemia group (median 0.72 vs. 0.83, *p* = 0.02). The cerebral hypoperfusion-specific CT-FFR cut-off value of 0.80 was independently associated with recurrent ischemic event (adjusted hazard ratio, 2.97 [95%CI: 1.32, 7.14]; *p* = 0.03). CT-FFR provided improved prognostic value beyond DS alone with the area under the curve value upon leave-one-out cross-validation (0.72 [95%CI: 0.64, 0.80] vs. 0.66 [95%CI: 0.57, 0.74]; *p* = 0.001), C-index (0.704 [95%CI: 0.613, 0.795] vs. 0.648 [95%CI: 0.573, 0.723]; *p* = 0.002) and categorical net reclassification improvement index (0.264 [95%CI: 0.096, 0.479]; *p* = 0.03).

**Conclusion:**

A CT-FFR threshold specific to CTP-based hypoperfusion may achieve improved value compared to DS alone for predicting recurrent ischemic event within 1 year in patients with symptomatic MCA stenosis.

## Introduction

Intracranial arterial stenosis (ICAS) as one of the main pathogeneses of ischemic stroke or transient ischemic attack in Asian population (1), is associated with a high risk of recurrent stroke and death. Despite the implementation of optimal medical treatment (OMT), the rate of recurrent stroke in ICAS patients within 3 months, 1 year and 2 years are up to 11.3, 20.0 and 25.0%, respectively (2–4), which suggest the need of more aggressive treatment for these high-risk individuals. Endovascular therapy (EVT) has emerged as a potential treatment option for ICAS. However, previous studies have not demonstrated a clear superiority of EVT over OMT (5, 6). With the rapid development of interventional technology, recent research has indicated a reduced perioperative event rate associated with endovascular stenting (7, 8), thereby increasing the interest in intracranial EVT within the field of neurology. There have been concerns that the current preoperative assessment of ICAS solely based on anatomical evaluation of cerebral artery stenosis, particularly the severity of luminal stenosis on CT angiography (CTA), has prevented effective stratification of patients for appropriate therapies (4). Appropriate non-invasive functional imaging evaluation techniques are urgently required for guiding optimal management of ICAS.

Over the past decade, noninvasive functional imaging evaluation techniques in the field of cardiology have been developed, especially based on computational fluid dynamics. The non-invasive CT-derived fractional flow reserve (CT-FFR) has significantly advanced CT technology from anatomy to functional assessment, demonstrating substantial value in guiding the clinical management of coronary artery disease (9, 10). Inspired by the success of coronary CT-FFR, there is an urgent need to introduce this technique to investigate the functional implications of ICAS (11). Nonetheless, the application of cerebral CT-FFR for the functional assessment of ICAS is rarely reported (12, 13). The extremely limited clinical use of invasive cerebral FFR (the reference standard) is primarily due to the inherent risk of the invasive FFR procedure, operational complexity, the high cost of the pressure guidewire, and, importantly, the lack of a definitive FFR cutoff value, which hinder the development of noninvasive cerebral CT-FFR (14, 15). CT perfusion (CTP) is capable of accurately reflecting the brain tissue perfusion, and has been shown to predict recurrent ischemic stroke in patients with symptomatic chronic carotid or middle cerebral artery occlusion (16). One study demonstrated that lower CT-FFR values were associated with downstream hypoperfusion as observed on cerebral CT perfusion (17). However, to the best of our knowledge, there is a lack of evidence to establish a clinically applicable intracranial artery CT-FFR cut-off value for detecting cerebral hypoperfusion, and whether this CT-FFR cut-off value is related to the recurrent ischemic risk in patients with symptomatic middle cerebral artery (MCA) stenosis.

Therefore, the aims of this study were (1) to define CT-FFR cut-off value for detecting brain tissue ischemia with CTP findings as reference standard; (2) to investigate the associations between CT-FFR and the risk of recurrent ischemic event, and (3) to explore the potential association between CT-FFR and clinical management strategy in patients with symptomatic MCA stenosis.

## Methods

### Study subjects

This retrospective study enrolled consecutive patients with ischemic stroke or transient ischemic attack admitted to Jinling Hospital from September 2014 to June 2021. The following inclusion criteria were used: (1) patients aged 18 years or older; (2) patients undergoing four-dimensional CTA (4D-CTA) examination within one month of stroke or transient ischemic attack onset; (3) the index ischemic event was attributed to unilateral atherosclerotic MCA stenosis (30–99%) at M1 segment diagnosed by stroke neurologists. Patients were excluded if they had (1) coexistent ipsilateral extracranial arterial stenosis of greater than 50%; (2) occluded lesions of the target artery; (3) previously received cerebrovascular surgery or interventional therapy; and (4) poor image quality of 4D-CTA, defined as the presence of severe motion artifacts, inadequate contrast opacification (<150 HU), poor signal-to-noise ratio. A detailed review of electronic health records was conducted to record the patients’ baseline characteristics. The study was approved by the Institutional Review Board of Hospital, which waived the requirement for specific patient informed consent due to the retrospective study design.

### CT protocols and image analysis

All 4D-CTA examinations were performed on a dual-source CT scanner (Somatom Definition FLASH, Siemens Healthcare, Forchheim, Germany) to provide dynamic imaging of whole-brain vasculature and perfusion. The dual-source CT scanner with the adaptive four-dimensional spiral scan mode allowed time-resolved scanning of areas larger than the detector width by continuous periodic table motion between the two end positions. The standard protocols contain 21 whole-brain passes divided into two parts. The first part of the scan includes 17 passes at a rate of 1.5 s every pass, while the second part consists of 4 passes at a rate of 3 s every pass, giving a total scan time of 37.5 s. For all patients, 30 mL of iodinated contrast agent (iopromide, Ultravist 300 mg I/mL, Bayer Schering Pharma, Berlin, Germany) was intravenously injected into the antecubital vein with a flow rate of 5.0 mL/s, followed by 20 mL of saline solution with the same flow rate. Detailed 4D-CTA protocol is listed in Supplementary Table 1.

All 4D-CTA source data were transferred to a commercial workstation (MultiModality Workplace; Siemens Healthcare, Forchheim, Germany) and processed at 5-mm thickness for perfusion image analysis. 4D Noise Reduction and Motion Correction techniques (Siemens Healthcare, Forchheim, Germany) were used to ensure image quality. The vessel segmentation thresholds were reviewed with automatic arterial and venous vessel identification, and segmentation of bone, CSF, and vessels were also applied. Additionally, grayscale and color-coded perfusion parameter maps for cerebral blood flow (CBF), cerebral blood volume (CBV), time to peak (TTP), mean transit time (MTT) and time-to-maximum of the tissue residue function (Tmax) were calculated for quantitative evaluation. Six regions of interest (4-cm-length) were manually and symmetrically drawn in bilateral MCA territories on CTP maps at the centrum semiovale, corona radiata, and basal ganglia levels (Supplementary Figure 1) (18). Relative values of CBF (rCBF), CBV (rCBV), MTT (rMTT), and TTP (rTTP) of the ipsilesional MCA territory were calculated as the ratio of ipsilesional and contralesional absolute values averaged from the regions of interest, and cerebral hypoperfusion was defined as MTT > 6.5 s (16).

With the same data set, the CTA of the whole intracranial vessel was reconstructed with slice thickness of 0.75 mm, and all CTA images were reconstructed from the peak arterial phase of the time-attenuation curve for the quantitative assessment of MCA stenosis severity on dedicated CT post-processing workstation (SyngoVia VB20, Siemens). The degree of stenosis was calculated according to The Warfarin-Aspirin Symptomatic Intracranial Disease Study (WASID) method: diameter stenosis (DS) = [1 - stenosis diameter / normal proximal segment diameter] × 100%, while stenosis severity was divided into mild (< 50%), moderate (50–69%) and severe (70–99%) (19).

Due to the importance of leptomeningeal collateral status in prognostic evaluation of stroke patients, leptomeningeal collateral status of MCA territories was assessed on the basis of a 6-point ordinal scale (15), and the peak arterial phase, early venous phase, and late venous phase phases of the CTP original images were chosen to grade collateral scores according to the following criteria: (1) grade 5: no delay and normal or increased prominence of vessels; (2) grade 4: delay of one phase in filling in of peripheral vessels, but prominence and extent are the same; (3) grade 3: delay of two phases in filling in of peripheral vessels or delay of one phase with the prominence or number of vessels significantly decreased; (4) grade 2: delay of two phases in the filling of peripheral vessels and decreased extent or one phase delay and some ischemic regions without vessels; (5) grade 1: just a few vessels visible in any phase within the occluded vascular territory; (6) grade 0: no vessels visible in any phase within the ischemic vascular territory. Collateral status was dichotomized into good (score of 4, 5) or poor (score of 0–3) (20).

Two experienced neuroradiologists (Z.Y. and C.Z. with 3- and 5-year experience in 4D-CTA interpretation) who were blinded to clinical information, evaluated the stenosis degree and collateral circulation status independently, any disagreement between two investigators were resolved by consensus reading.

### CTA-based computational fluid dynamics simulation

#### Modeling and meshing

Based on the CTA source images, the patient-specific geometry, including the internal carotid artery (ICA), the M1 segment of the MCA, and the A1 segment of anterior cerebral artery (ACA), was first segmented using Mimics software (Materialise Inc., Leuven, Belgium), and then the stenosis site and its surrounding vessels were extracted and optimized on the basis of the segmented mask (Supplementary Figure 2). The extracting of fluid domain from the above 3D model and volume meshing was preformed using the open-source SimVascular software package (SimVascular Development Team, US) (21). Considering the complexity of the geometry, we used the unstructured tetrahedral cell for domain discretization, and the mesh was finer at the stenosis site where the flow field was of greater interest.

#### Numerical methods

Blood was modeled as an incompressible Newtonian fluid with a density of 1,060 kg·m^3^ and a dynamic viscosity of 0.004 Pa·s. The temperature and gravity changes were ignored in the process of numerical simulation, and the blood flow was thought as laminar flow. The 3D incompressible Navier–Stokes equations were used to describe the momentum conservation of blood flow.

In addition, the vessel was assumed as rigid, and non-slip boundary condition was assigned to the wall, and the steady flow was adopted. Due to the lack of measurements of arterial blood flow in patients, the corresponding arterial flow value in the references was used as the inlet boundary condition. At the outlets of the efferent arteries, the lumped parameter model was coupled with the 3D arterial model to simulate the distal resistance. The resistance of each outlet of the model ( $R_{i}$ ) could be calculated by using the following Equations 1, 2:


$$R_{\text{total}}=\frac{P_{\text{mean}}}{Q}$$ (1)

$$R_{i}=\frac{\sum_{j}A_{j}}{A_{i}}R_{\text{total}}$$ (2)

In Equation 1, $R_{\text{total}}$ is the total resistance of all outlets of the model, $P_{\text{mean}}$ is the average pressure at the inlet with the patient’s average blood pressure because of the lack of measurements, Q is the flow of the inlet. In Equation 2, $R_{i}$ and $A_{i}$ are the resistance and area of the i-th outlet, respectively. $\sum_{j}A_{j}$ is the total area of all outlets of the model. In order to ensure the reliability of simulation results, each model in this study was solved for 3 cardiac cycles (3 s) with a time step of 0.01 s, and the calculation results of the third cardiac cycle (2–3 s) were adopted.

#### CT-FFR measurement

All CT-FFR measurements were conducted utilizing the open-source Paraview software (version 5.5.2, Kitware Inc., New York), which facilitates interactive scientific visualization (22). The measurement site was chosen at 1 cm distal to the stenosis, if the distal trunk of the stenosis was insufficiently long for 1 cm, the normal segment proximal to the MCA-M1 bifurcation was used as an alternative site (23). A single investigator (Z.Y., with three years of experience in computational fluid dynamics modeling) performed the CT-FFR assessments for all cases twice in succession with a four-week interval, and the average of the two measurements was considered the final result.

#### Treatment, follow-up procedures and outcome

All patients received contemporary optimal OMT or EVT (including both angioplasty and stenting, either alone or in combination) in accordance with up-to-date clinical guidelines after undergoing baseline 4D-CTA (24). The National Institutes of Health Stroke Scale (NIHSS) score was recorded to assess the poststroke clinical course during hospitalization, and the neurological deterioration was defined as an increase in the NIHSS score by ≥4 points (25). From the time of 4D-CTA examination, complete 1-year follow-up data were obtained for all patients by neurology outpatient clinic or telephone. Recurrent ischemic event was defined as a composite of recurrent ischemic stroke in the same territory and recurrent transient ischemic attack relevant to ischemia in the same territory within 1 year. Recurrent ischemic stroke in the same territory was confirmed by new infarct on CT or magnetic resonance imaging upon stroke recurrence. Transient ischemic attack was defined as a transient episode of neurological dysfunction caused by focal brain ischemia without acute infarction in cases with CT or magnetic resonance imaging examination or the transient neurological dysfunction completely resolved within 24 h determined by neurologists according to the symptoms if there was no brain imaging available.

### Statistical analysis

Continuous variables were expressed as mean±standard deviation or median and interquartile range (IQR), whereas categorical variables were expressed as number and percentage (%). Univariate comparisons between two groups were performed using independent t tests, Mann–Whitney tests or χ^2^ test. The inter- and intra-observer agreements were assessed using intraclass correlation coefficient (ICC) and Cohen’ kappa test. The Kappa value or ICC was interpreted as fair (0.20–0.40), moderate (0.41–0.60), good (0.61–0.80), or excellent (0.81–1.00) agreement (26). The area under the curve (AUC) derived from receiver-operating characteristic curve of CT-FFR was calculated for discriminating cerebral hypoperfusion from normal perfusion. The optimal threshold of CT-FFR was calculated according to the Youden index. An internal validation for the optimal CT-FFR cut-off value was performed using bootstrapping with 1,000 resamples, and each 0.05-unit strata was set as independent threshold in the CT-FFR range of 0.50 to 0.90. The Fisher’s precision probability test was used to compare the diagnostic characteristics, and a Delong test was performed to compare AUC. The correlation between CT-FFR and perfusion parameters was evaluated by Spearman correlation coefficient. Univariable and multivariable logistic regression analyses were performed to identify the independent factors associated with cerebral hypoperfusion.

Dichotomized CT-FFR was analyzed in separate multivariable Cox proportional hazards regression models for the recurrent ischemic event, adjusting for admitting diagnosis, collateral status and other baseline variables with *p* value less than 0.05 in univariable comparisons. Prediction model performance was assessed by using Harrell C-index and net reclassification improvement (NRI). Kaplan–Meier curves were constructed for the cumulative probabilities of the recurrent ischemic event. A univariable analysis was performed on clinically relevant variables with inclusion into a multivariable model if the *p* value was less than 0.10 to obtain the relationship of the recurrent ischemic event with each per 0.05-unit decrement of CT-FFR and 10%-unit increase of DS (27). A value of *p* < 0.05 was considered statistically significant. All analyses were conducted by using R programming language (version 3.6.1; R Foundation for Statistical Computing, Vienna, Austria), SPSS Statistics (version 26.0.0, IBM SPSS Statistics, Armonk, New York) and MedCalc (version 20.0.1, MedCalc Software, Ostend, Belgium).

## Results

### Patient characteristics

**Figure 1:**
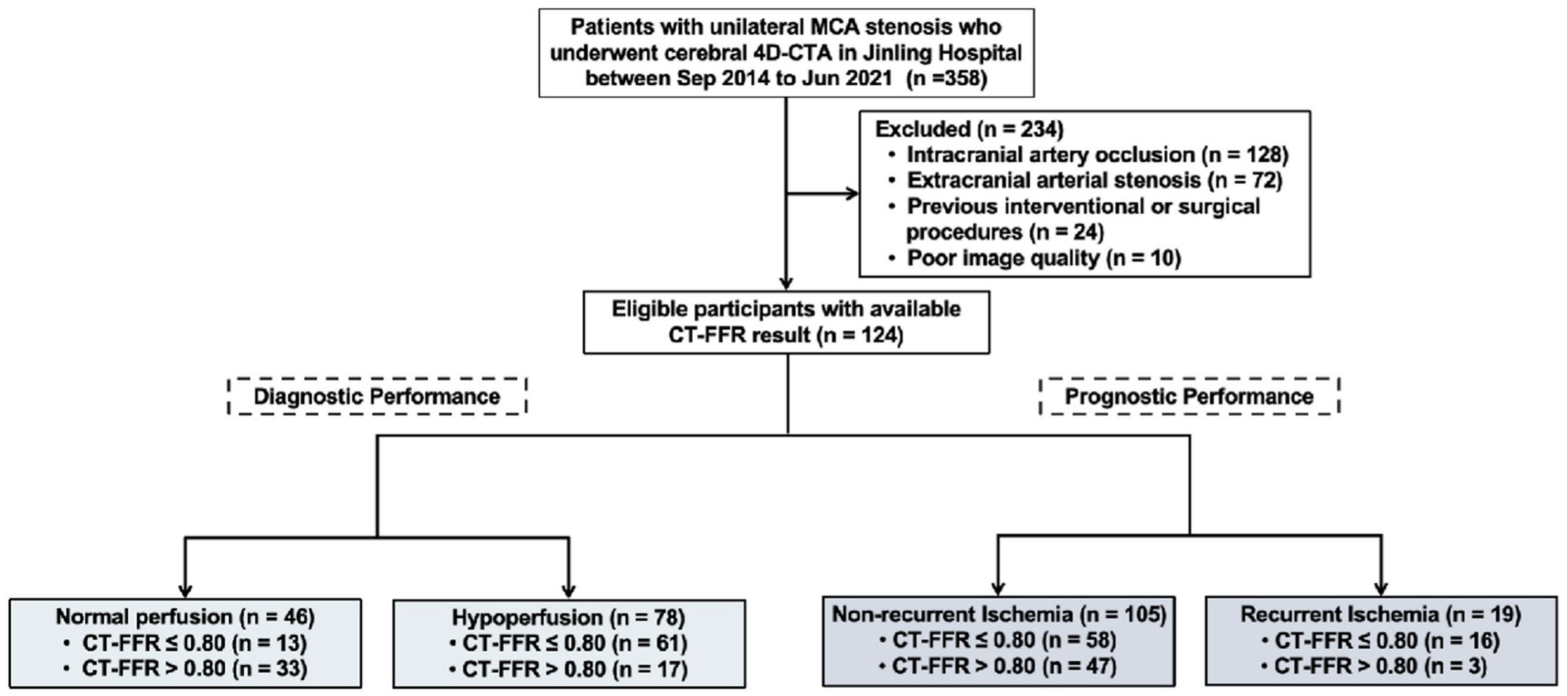
Flow chart of the study. MCA, middle cerebral artery; 4D-CTA, four-dimensional computed tomographic angiography; CT-FFR, CT-derived fractional flow reserve.

Patient characteristics are summarized in Table 1. Of 358 patients screened, 234 participants were excluded due to intracranial artery occlusion (*n* = 128), extracranial arterial stenosis (*n* = 72), previous interventional or surgical procedures (*n* = 24), or poor image quality (*n* = 10). Finally, 124 eligible patients (median age, 58 years; interquartile range, 51–63 years; 96 men) were included in this study (Figure 1). The admitting diagnosis was ischemic stroke in 87 patients (70.2%) and transient ischemic attack in 37 patients (29.8%). Most patients had severe MCA stenosis on baseline 4D-CTA (92/124, 74.2%). Of these, 31 patients subsequently underwent EVT after a median interval of 3 days (IQR: 1–7 days) from 4D-CTA to treatment, and no patient experienced neurological deterioration. The median value of CT-FFR was 0.78 (IQR: 0.73, 0.81), and 19 (15.3%, 19/124) patients with recurrent ischemic event presented lower CT-FFR than those without recurrent ischemia (84.7%, 105/124) (0.72 [IQR: 0.65, 0.77] vs. 0.83 [IQR: 0.76, 0.88]; *p* = 0.02). The intra-observer agreement for CT-FFR (ICC = 0.95), inter-observer agreement for assessment of DS (Kappa = 0.91), and collateral status (Kappa = 0.87) were all excellent.

**Table 1:** Baseline characteristics of patients stratified by recurrent ischemia.

Characteristics	Overall (*n* = 124)	Non-recurrent ischemia (*n* = 105)	Recurrent ischemia (*n* = 19)	*p* value
Age (y)	58 (51–63)	57 (50–62)	60 (52–69)	0.47
Male sex, *n* (%)	96 (77.4%)	82 (78.1%)	14 (73.7%)	0.90
Current smoker, *n* (%)	68 (54.8%)	59 (56.2%)	9 (47.4%)	0.47
Hypertension, *n* (%)	75 (60.5%)	64 (61.0%)	11 (57.9%)	0.80
Diabetes mellitus, *n* (%)	21 (16.9%)	16 (15.2%)	5 (26.3%)	0.39
Systolic blood pressure (mmHg)	145 (132–157)	143 (135–152)	147 (134–160)	0.65
Diastolic blood pressure (mmHg)	80 (71–87)	81 (72–88)	79 (71–86)	0.46
Fasting blood glucose (mmol/L)	5.8 ± 1.0	6.0 ± 1.1	5.6 ± 1.2	0.31
Total cholesterol (mmol/L)	3.7 ± 0.9	3.7 ± 1.0	3.6 ± 0.8	0.73
HDL (mmol/L)	1.1 ± 0.4	1.1 ± 0.3	1.1 ± 0.4	0.90
LDL (mmol/L)	2.1 ± 0.8	2.1 ± 0.7	2.1 ± 1.0	0.87
Triglycerides (mmol/L)	1.5 ± 0.9	1.5 ± 0.8	1.6 ± 1.0	0.61
Admitting diagnosis, *n* (%)				0.85
Transient ischemic attack	37 (29.8%)	31 (29.5%)	6 (31.6%)	
Ischemic stroke	87 (70.2%)	74 (70.5%)	13 (68.4%)	
Time from onset to CT exam (d)	19 (10–25)	21 (15–29)	15 (6–21)	0.42
Treatment, *n* (%)				0.88
OMT	93 (75.0%)	79 (75.2%)	14 (73.7%)	
EVT	31 (25.0%)	26 (24.8%)	5 (26.3%)	
Anatomic severity of lesion, *n* (%)				0.34
Severe	92 (74.2%)	75 (74.3%)	17 (73.7%)	
Moderate	25 (20.2%)	23 (19.0%)	2 (26.3%)	
Mild	7 (5.6%)	7 (4.0%)	0 (0.0%)	
Cerebral hypoperfusion, *n* (%)	78 (62.9%)	64 (61.0%)	14 (73.7%)	0.29
MTT (s)	6.8 (5.5–7.6)	6.6 (5.9–7.2)	7.4 (6.6–8.6)	0.21
Good collateral status, *n* (%)	71 (57.3%)	62 (59.0%)	9 (47.4%)	0.34
CT-FFR	0.78 (0.73–0.81)	0.83 (0.76–0.88)	0.72 (0.65–0.77)	0.02
CT-FFR ≤ 0.80, *n* (%)	74 (59.7%)	58 (55.2%)	16 (84.2%)	0.03

HDL, high density lipoprotein; LDL, low density lipoprotein; OMT, optimal medical treatment; EVT, endovascular treatment; CT-FFR, CT-derived fractional flow reserve.

### Diagnostic performance of CT-FFR in predicting cerebral ischemia

Forty-six (37.1%) participants had normal cerebral perfusion and 78 (62.9%) had cerebral hypoperfusion in the ipsilateral MCA region (Supplementary Table 2). The mean CT-FFR value of the hypoperfusion group was significantly lower than that of normal perfusion group (0.73 ± 0.09 versus 0.84 ± 0.06; *p* < 0.001). Table 2 shows the results of the logistic regression analyses for predicting cerebral hypoperfusion. The multivariable logistic regression analysis results showed that hypertension (adjusted OR, 4.87; 95%CI: 1.52, 27.92; *p* = 0.03), collateral status (adjusted OR, 0.40; 95%CI: 0.31, 0.95; *p* = 0.04) and CT-FFR (adjusted OR, 5.92; 95%CI: 1.42, 41.12; *p* = 0.02) were independently associated with cerebral hypoperfusion in the target MCA terrority on CTP. Association of CT-FFR with cerebral perfusion can be found in Supplementary Tables 3, 4.

**Table 2:** Univariable and multivariable logistic regression models for predicting cerebral hypoperfusion on CT perfusion.

Variables	Univariable model		Multivariable model	
	OR (95%CI)	*p* value	OR (95%CI)	*p* value
Age (y)	0.97 (0.91–1.06)	0.59		
Gender (M vs. F)	0.52 (0.32–1.58)	0.32		
Current smoker (Y vs. N)	1.98 (0.72–13.60)	0.29		
Hypertension (Y vs. N)	5.14 (1.23–45.12)	0.04	4.87 (1.52–27.92)	0.03
Diabetes mellitus (Y vs. N)	0.82 (0.21–3.65)	0.41		
Stenosis severity (severe vs. non-severe)	1.37 (0.86–3.32)	0.06		
Collateral status (Good vs. Poor)	0.47 (0.29–1.07)	0.04	0.40 (0.31–0.95)	0.04
CT-FFR (≤ 0.80 vs. > 0.80)	6.37 (1.15–48.63)	0.03	5.92 (1.42–41.12)	0.02

M, male; F, female; Y, yes; N, No; CT-FFR, CT-derived fractional flow reserve.

**Figure 2:**
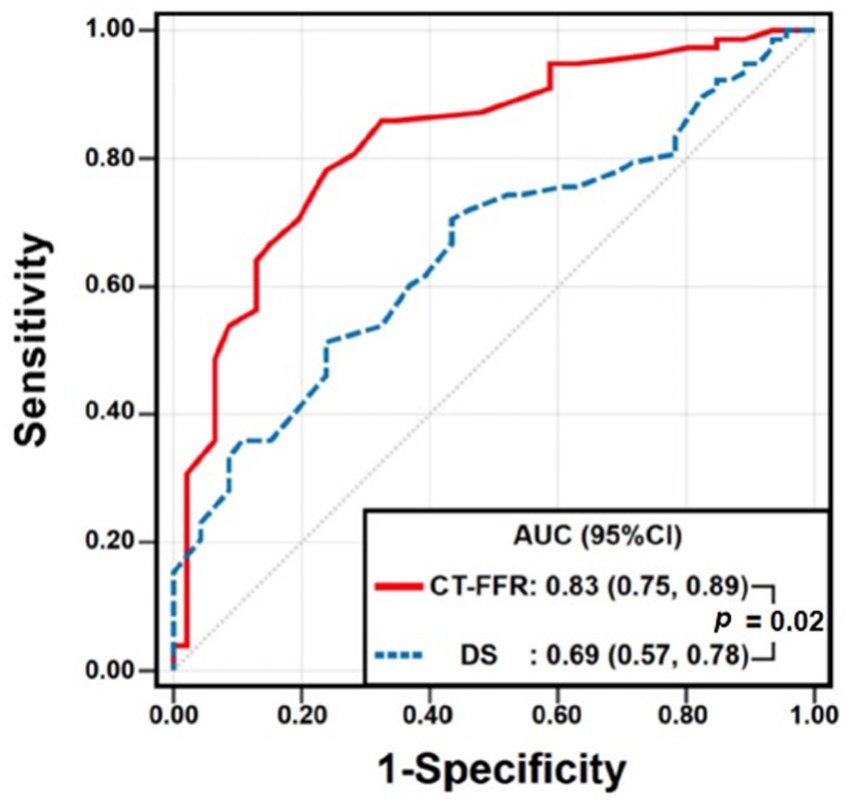
Receiver operating characteristics analysis shows the diagnostic performance comparison of CT-FFR and DS in predicting cerebral hypoperfusion. AUC, area under the curve; CT-FFR, CT-derived fractional flow reserve; DS, diameter stenosis.

**Figure 3:**
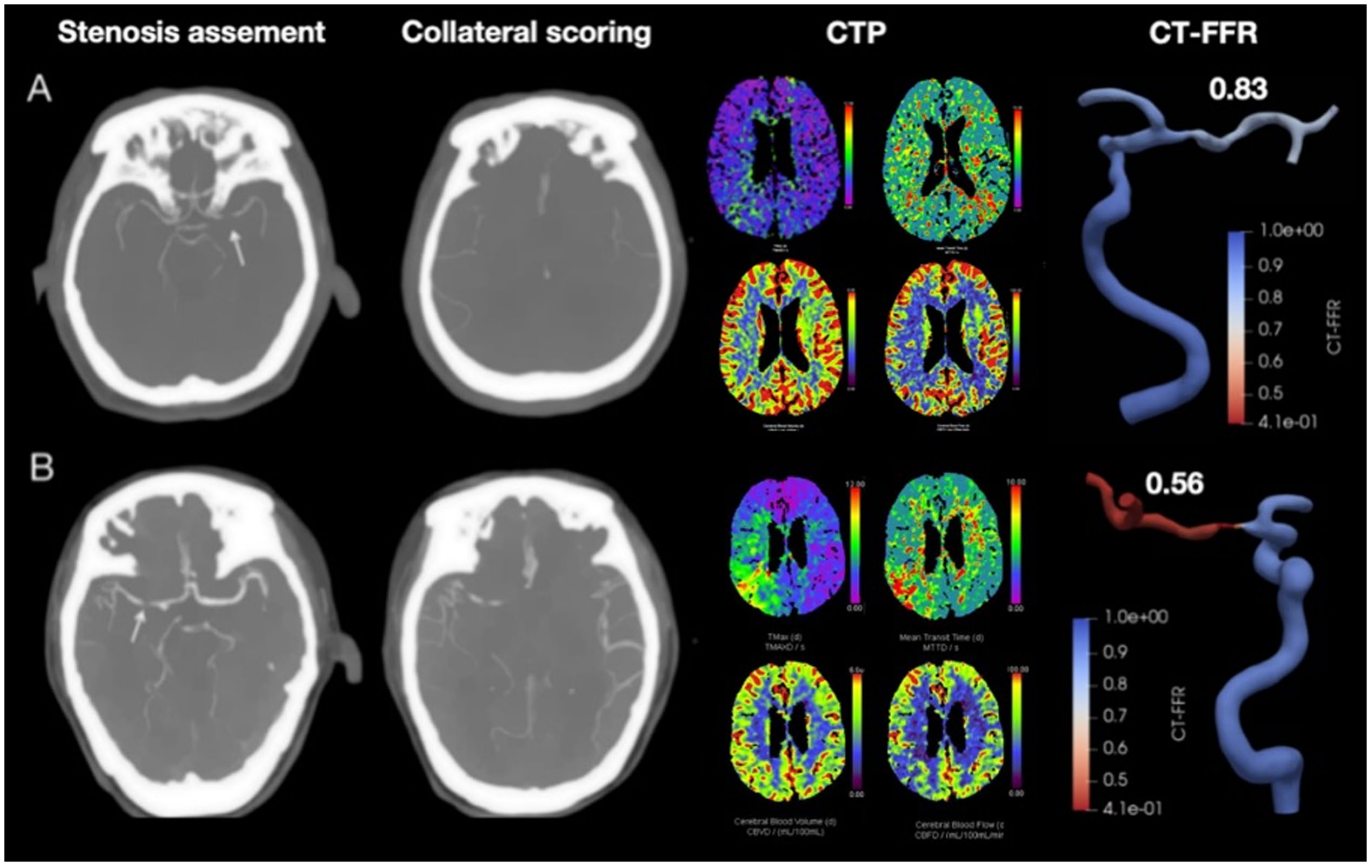
Examples of two patients with severe intracranial artery stenosis. Case 1 **(A)**: classified into the non-recurrent ischemia cohort. CT angiography (CTA) shows 75% stenosis at M1 segment of the left middle cerebral artery (white arrow) and normal prominence of pial arterial compared to contralateral hemispheres (collateral score = 5); CTP demonstrates normal cerebral perfusion, and the value of CT-FFR was 0.83. Case 2 **(B)**: classified into the recurrent ischemia cohort. Atherosclerotic plaque at M1 segment of the right middle cerebral artery results in 87% stenosis (white arrow). Multiphase CTA shows some ischemic regions without visualization of brain vessels (collateral score = 2). Cerebral hypoperfusion in the right temporo-parietal lobe on CTP (increase in Tmax and MTT, reduction in CBF, and normal CBV) and low CT-FFR (CT-FFR = 0.56) were shown. Tmax, time-to-maximum of the tissue residue function; MTT, mean transit time; CBF, cerebral blood flow; CBV, cerebral blood volume; CT-FFR, CT-derived fractional flow reserve.

From the ROC curve of CT-FFR and DS for cerebral ischemia prediction, the AUC of CT-FFR was significantly higher than that of DS (0.83 [95%CI: 0.75, 0.89] vs. 0.69 [95%CI: 0.57, 0.78], *p* = 0.02) (Figure 2), and the cut-off value of 0.79 showed the highest Youden index. Internal validation by 1,000 times Bootstrap resampling revealed that the 95%CI of optimal CT-FFR cut-off value was 0.72 to 0.82. Therefore, considering the above findings and clinical practicality, the integer 0.80 was determined as the optimal cut-off value. Additionally, the cut-off value of 0.80 showed the highest AUC with sensitivity of 79.5% (95%CI: 68.9, 87.8%), specificity of 73.9% (95%CI: 59.0, 85.7%), compared to the other cut-offs (range of 0.50 to 0.90, each 0.05-unit strata). The diagnostic performances of different dichotomized CT-FFR are shown in Supplementary Table 5. Two illustrative cases are shown in Figure 3.

### Prognostic value of CT-FFR

**Figure 4:**
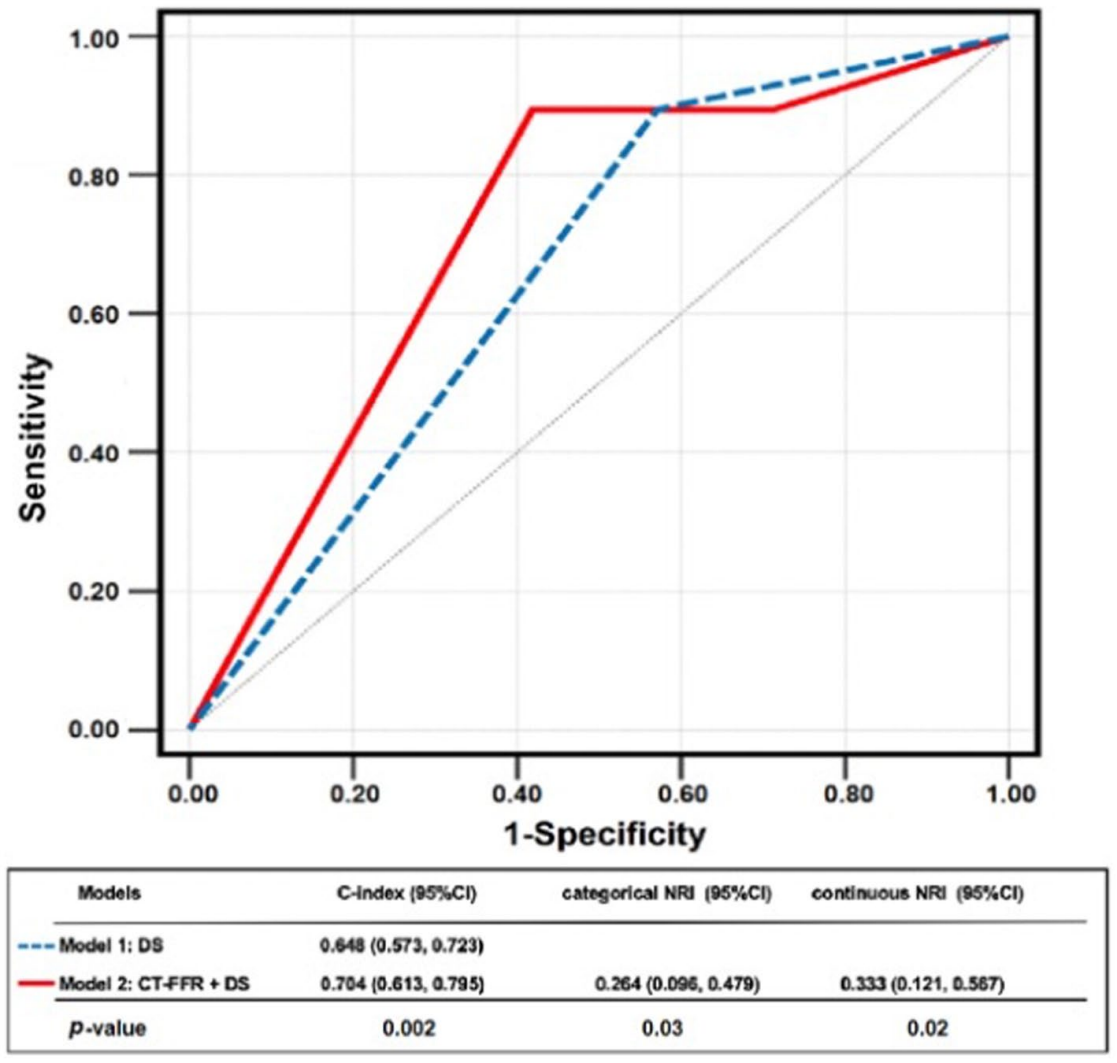
Improved prognostic value of CT-FFR over DS alone. The model with CT-FFR significantly increased discriminant and reclassification abilities for predicting recurrent ischemic event compared to the DS alone model. AUC, area under the curve; NRI, net reclassification improvement index.

Of 124 patients, 19 (15%) patients suffered from recurrent ischemic event during 1 year follow-up period after index 4D-CTA examination, the incident rate of CT-FFR ≤ 0.80 group (16/74, 21.6%) was significantly higher than CT-FFR > 0.80 group (3/50, 6.0%) (*p* = 0.03). In multivariable Cox regression model, CT-FFR ≤ 0.80 (adjusted HR: 2.97 [95%CI: 1.32, 7.14]; *p* = 0.03) and DS ≥ 70% (adjusted HR: 2.18 [95%CI: 1.39, 3.67]; *p* = 0.04) were independent predictive factors for the recurrent ischemic event (Table 3). Changes in risk prediction of DS and CT-FFR are shown in Figure 4. CT-FFR provided significantly improved prognostic value beyond DS alone with C-index (0.704 [95%CI: 0.613, 0.795] vs. 0.648 [95%CI: 0.573, 0.723]; *p* = 0.002), categorical net reclassification improvement index [NRI] (0.264; 95%CI: 0.096, 0.479; *p* = 0.03) and continuous NRI (0.333; 95%CI: 0.121, 0.567; *p* = 0.02).

**Table 3:** Univariable and multivariable COX regression models for recurrent ishchemic event.

Variables	Univariable model		Multivariable model	
	HR (95%CI)	*p* value	HR (95%CI)	*p* value
Age (y)	1.02 (0.94–1.09)	0.79		
Gender (M vs. F)	0.73 (0.53–1.37)	0.47		
Admitting diagnosis (ischemic stroke vs. transient ischemic attack)	0.88 (0.72–1.03)	0.72		
Hypertension (Y vs. N)	1.23 (0.83–1.72)	0.08		
Diabetes mellitus (Y vs. N)	0.98 (0.84–1.14)	0.55		
Stenosis severity (severe vs. non-severe)	2.76 (1.45–5.87)	0.03	2.18 (1.39–5.67)	0.04
Collateral status (Good vs. Poor)	0.89 (0.57–1.09)	0.48		
Cerebral perfusion (hypoperfusion vs. normal perfusion)	1.12 (0.79–1.54)	0.17		
Treatment (OMT vs. EVT)	1.19 (0.72–1.66)	0.20		
CT-FFR (≤ 0.80 vs. > 0.80)	3.38 (1.62–7.78)	0.01	2.97 (1.32–7.14)	0.03

M, male; F, female; Y, yes; N, No; OMT, optimal medical treatment; EVT, endovascular treatment; CT-FFR, CT-derived fractional flow reserve.

### Association of continuous CT-FFR with clinical outcomes

The incidence and relative HRs of the recurrent ischemic event per 0.05-unit decrement of CT-FFR and 10%-unit increase of DS are illustrated in Supplementary Figure 3. There were increasing trends in 1-year rate of recurrent ischemic event with decreasing CT-FFR (*p* for trend = 0.001) and increasing DS (*p* for trend = 0.04). After adjusting for potential confounders using multivariable Cox regression model, each 0.05 - unit lower CT-FFR (adjusted HR, 2.37; 95%CI: 1.26, 4.89; *p* = 0.01) was significantly associated with a higher incidence of recurrent ischemic event, while the adjusted HR of 10% - unit higher DS was 1.41 (95%CI: 1.15, 2.30; *p* = 0.03).

### Physiology versus anatomy assessment matrix

**Figure 5:**
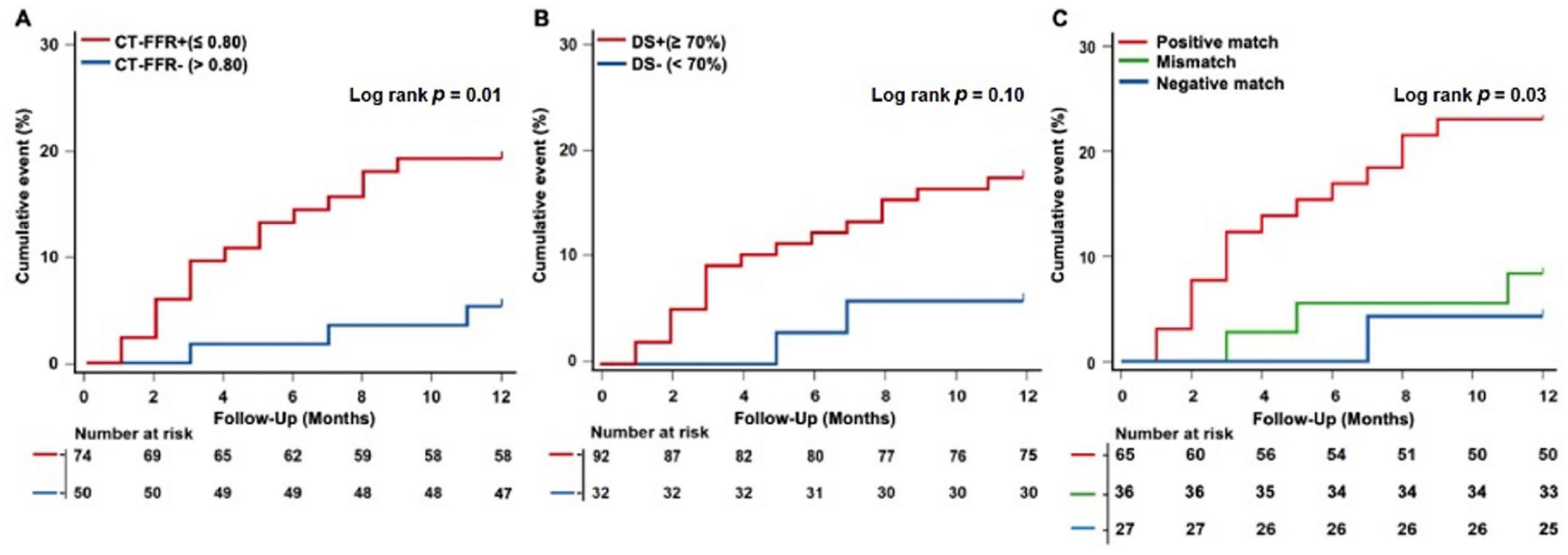
Kaplan–Meier curves show prognostic value of CT-FFR. **(A,B)** Curves for recurrent ischemic event within 1 year stratified according to CT-FFR and DS. **(C)** Estimates stratified by the presence (+) or absence (−) of low CT-FFR and severe diameter stenosis (≥70%). CT-FFR, CT-derived fractional flow reserve; DS, diameter stenosis.

In this study, we used CT-FFR ≤ 0.80 and DS > 70% as the thresholds for positive diagnosis, and, respectively, defined the positive group and the negative group (CT-FFR: ≤ 0.80 vs. > 0.80; DS: > 70% vs. ≤70%). No significant difference was found in the incidence of recurrent ischemic event between CT-FFR positive (CT-FFR+) group and DS positive (DS+) group (21.6% vs. 18.5%, *p* = 0.614). Further stratifying by CT-FFR and DS, the match and mismatch CT-FFR and DS matrix are shown in Supplementary Figure 4. The recurrent event rate of positive match group [CT-FFR(+) / DS(+)] was 23.07% (15/65), which was significantly higher than those of mismatch group [CTFFR(+) / DS(−): 1/9, 11.1%; CTFFR(−) / DS(+): 2/27, 7.4%] and negative match group [CTFFR(−) / DS(−): 1/23, 4.3%] (*p* for trend = 0.01). Figure 5 shows the Kaplan–Meier curves for the cumulative probabilities by CT-FFR, DS and their combinations. A significantly higher risk of the recurrent ischemic event was demonstrated in the group of CT-FFR ≤ 0.80 (HR, 3.03; 95%CI: 1.21, 7.56; log rank *p* = 0.01). In comparison, the HR value for the group of severe DS (HR, 2.32; 95%CI: 0.84, 6.44; log rank *p* = 0.10) was not statistically significant. Furthermore, the positive match group had higher risk compared with mismatch group (HR, 3.05; 95%CI: 1.08, 8.57) and negative mismatch group (HR, 5.93; 95%CI: 1.80, 19.62) (log rank *p* = 0.03).

### Potential impact of CT-FFR on treatment strategy

In order to explore the potential impact of CT-FFR on the following treatment strategy, we hypothesized that the patients with DS > 70% or CT-FFR ≤ 0.80 were required to undergo EVT. CT-FFR resulted in revision of the recommended management strategy in 29.0% (36/124) of patients when compared with initial CTA-based management strategy (Supplementary Figure 5). Of 74.2% (92/124) patients recommended to receive EVT based on CTA, CT-FFR reclassified 29.3% (27/92) to OMT. Of these patients with cancelation of EVT recommended by CT-FFR, 61.1% (20/27) patients did not undergo EVT in the real treatment decision with a low incidence rate of the recurrent ischemic event (10.0%, 2/20). Thus, the recommended EVT rate could be reduced by 14.5% (from 74.2% [92/124] to 59.7% [74/124]) based on CT-FFR results compared with the CTA-based strategy, and 90% (18/20) patients recommended to delay EVT by CT-FFR results and actually received OMT had no recurrent ischemic event within 1 year of follow-up. Among 93 patients who actually received OMT alone, 14 had recurrent ischemic event (10 in the positive match group, 3 in the mismatch group, and 1 in the negative match group). Furthermore, within the positive match group, the incidence of recurrent ischemic events was lower in EVT patients than in OMT patients (16.1% vs. 21.3%).

## Discussion

In this study, we demonstrated that a CT-FFR cut-off value of 0.80 achieved superior diagnostic performance compared with anatomical stenosis in distinguishing cerebral hypoperfusion from normal perfusion. Notably, a CT-FFR value of ≤ 0.80 may serve as a novel independent risk factor for predicting recurrent ischemic events within one year, and provides significantly improved prognostic value beyond stenosis severity alone. Moreover, CT-FFR may be associated with clinical decision-making in patients with symptomatic intracranial atherosclerosis (ICAS). These findings suggest a potential role for CT-FFR in functional assessment, risk stratification, and exploratory associations with treatment selection for patients with ICAS.

In the field of coronary artery disease, CT-FFR has been shown to reduce unnecessary invasive coronary angiography and adverse cardiovascular events, and is recommended by guidelines to guide coronary revascularization (28, 29) Although CT-FFR has achieved considerable success in the functional assessment of coronary artery disease, it is less used in cerebral artery disease due to the limited conduction of invasive FFR clinical research, resulting in no FFR cut-off value as a reference standard for routine clinical work (4). Cerebral perfusion based on CTP should be considered the optimal reference standard for evaluating brain tissue ischemia, while the ultimate aim of CT-FFR analysis is to indirectly evaluate the ischemia of downstream tissues (30). CTP as a commonly used method to reflect cerebral perfusion can effectively diagnose brain ischemia, and has been used to guide therapeutic decision-making in acute ischemic stroke (31, 32). Based on CTP-defined brain ischemia, this study proposes a rational cutoff value of CT-FFR ≤ 0.80 with high diagnostic performance for discriminating cerebral hypoperfusion. We demonstrated the improved value of CT-FFR ≤ 0.80 in predicting recurrent ischemic events in patients with ICAS compared to DS alone. The combination of positively matched CT-FFR and DS was associated with the highest rate of recurrent ischemic events. Several studies have suggested the feasibility of cerebral CT-FFR in patients with ICAS (12, 13, 33, 34). Leng et al. (33) used the median translesional pressure ratio value of 0.94 as to dichotomize the patients, which indicated that low translesional pressure ratio (<0.94) was independently associated with the risk of recurrent stroke. Recently, Tian et al. (34) developed a nomogram model that integrates hemodynamic parameters, including the translesional pressure ratio, along with other risk factors, to effectively stratify patients with intracranial atherosclerosis (ICAS) based on their risk of recurrent stroke. However, these studies were either case reports (13) or case series (12), or no definitive CT-FFR cut-off value proposed and validated in recurrent risk stratification (33, 34). Consequently, this study is crucial in resolving this fundamental issue within the field of cerebral CT-FFR,

Although advancements in intracranial stenting technology have significantly influenced the efficacy of endovascular treatment (EVT), a major challenge remains due to the lack of appropriate preoperative imaging assessment techniques to identify high-risk patients who would benefit most from aggressive treatment and management (4, 11). From the further analysis of CT-FFR on management strategies in our study, the recommend EVT rate could be reduced by 14.5%, and most patients who were recommended to OMT by CT-FFR were safe within 1 year of follow-up. Among patients reclassified from EVT to OMT, 82.0% patients did not undergo EVT in the real treatment decision. This indicates that CT-FFR has the potential to discriminate the low-risk patients, who are more likely to safely delay EVT. Patients who were recommended for delayed EVT by CT-FFR and actually only received OMT had low recurrent ischemic event rate within 1 year. However, we noted that patients with severe stenosis and positive CT-FFR still carried a relatively high risk of recurrent ischemia after successful EVT, which is similar to the results of previous studies (5, 6). Further studies with the contemporary EVT techniques can partially resolve this issue.

There were some limitations in our study. First, we only recruited patients with symptomatic ICAS at the unilateral M1 segments of the MCA. Therefore, generalization of our study findings may be limited by the inherent selection bias. Second, this retrospective observational study was performed in a single-center with relatively small sample size, the diagnostic and prognostic performance of CT-FFR need to be validated by prospective studies with large sample size. Third, we used CTP rather than invasive FFR as reference standard to investigate the CT-FFR diagnostic performance because CTP has been widely acknowledged to well evaluate brain tissue ischemia. Fourth, due to the lack of patient-specific flow data, the inflow and outflow boundary condition values of computational fluid dynamics model in this study using generic values were derived from literature, which may affect the accuracy of simulation. Further studies should assess patient-specific physiological parameters using phase-contrast MRI, Doppler ultrasound, or other relevant modalities. Finally, the value of CT-FFR for treatment selection requires further exploration and validation in prospective studies. The ultimate goal of clinical validation should be to investigate whether CT-FFR guided interventions, such as intracranial stenting, improve patient outcomes (15). Taken together, future prospective studies with large sample size are needed to further demonstrate and extend our study findings.

In conclusion, CT-FFR is potentially useful in evaluating functional ischemia of ICAS with excellent repeatability and diagnostic performance. Cerebral CT-FFR with cut-off value of 0.80 had an excellent ability to recognize cerebral hypoperfusion on CTP and identify high risk population of recurrent ischemic event within 1 year in patients with symptomatic MCA stenosis, and may have a potential clinical utility for helping clinical decision making.

## Data Availability

The raw data supporting the conclusions of this article will be made available by the authors, without undue reservation.
