# Supplementary figures and images for: CT - derived fractional flow reserve can predict recurrent ischemia in patients with MCA stenosis
Source: Front Neurol. 2026 Jun 17;17:1838968. doi: 10.3389/fneur.2026.1838968 (PMC13318665; doi:10.3389/fneur.2026.1838968)

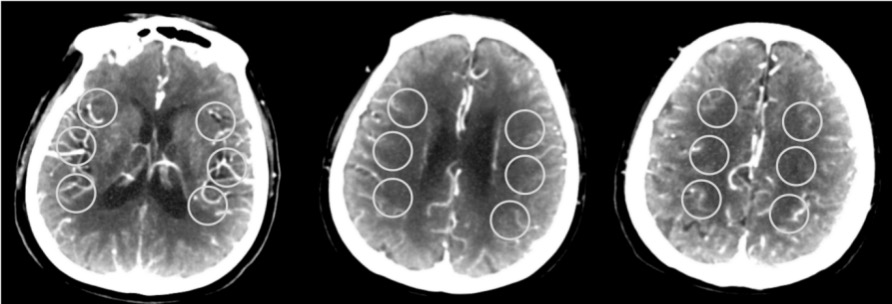

Supplement: Supplementary file 1 [file Image_1.TIFF]

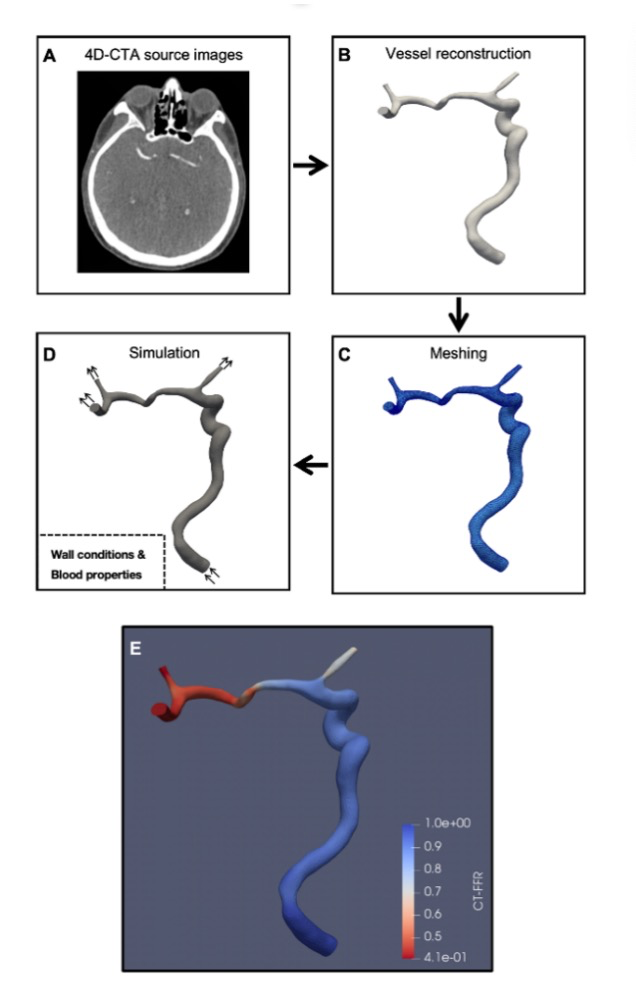

Supplement: Supplementary file 2 [file Image_2.TIFF]

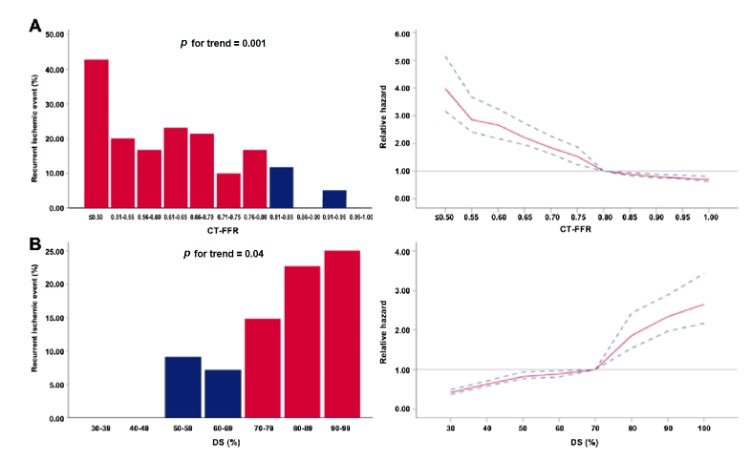

Supplement: Supplementary file 3 [file Image_3.TIFF]

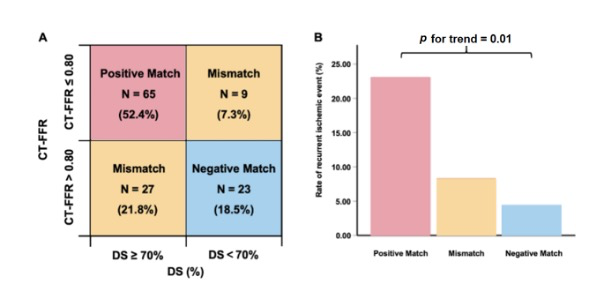

Supplement: Supplementary file 4 [file Image_4.TIFF]

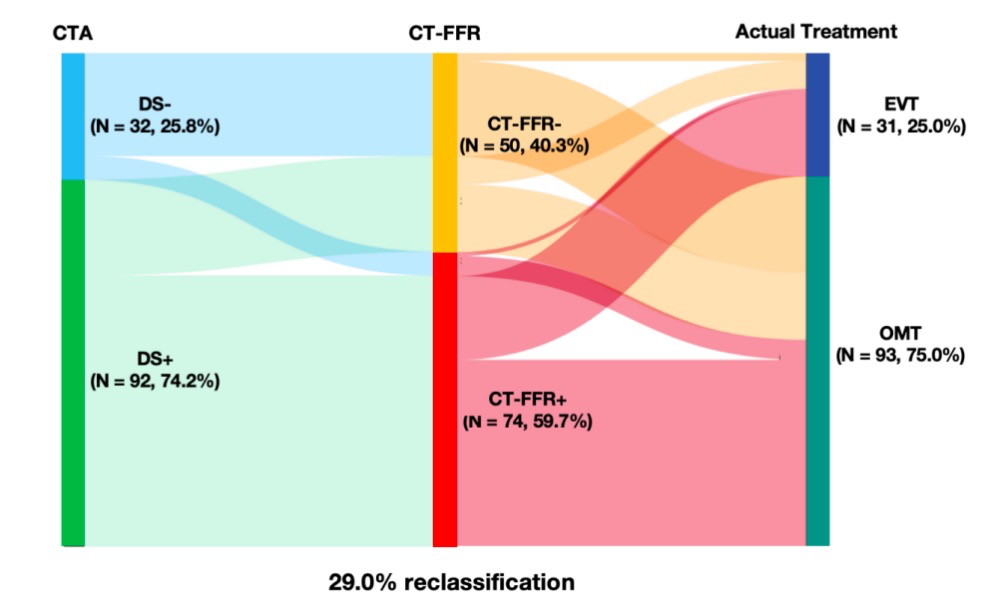

Supplement: Supplementary file 5 [file Image_5.TIFF]
