# Supplementary material for: CT - derived fractional flow reserve can predict recurrent ischemia in patients with MCA stenosis
Source: Front Neurol. 2026 Jun 17;17:1838968. doi: 10.3389/fneur.2026.1838968 (PMC13318665; doi:10.3389/fneur.2026.1838968)
Supplement: Supplementary file 6 [file Table_1.DOCX]

**Supplemental Materials**

**Association of CT-FFR with Cerebral Perfusion**

Compared with the group of CT-FFR > 0.80, more participants with CT-FFR ≤ 0.80 showed the hypoperfusion in the target MCA territory on CTP (82.4% [95%CI: 72.2%, 89.4%] vs 46.0% [95%CI: 33.0%, 59.6%]; *p* < 0.001). **Supplemental Table 3** shows the results of the logistic regression analyses for predicting cerebral hypoperfusion. The multivariable logistic regression analysis results showed that hypertension (adjusted OR, 4.87; 95%CI: 1.52, 27.92; *p* = 0.03), collateral status (adjusted OR, 0.40; 95%CI: 0.31, 0.95; *p* = 0.04) and CT-FFR (adjusted OR, 5.92; 95%CI: 1.42, 41.12; *p* = 0.02) were independently associated with cerebral hypoperfusion in the target MCA territory on CTP. We also found CT-FFR was mildly or moderately correlated with rTTP (r = -0.49; *p* < 0.001), rMTT (r = -0.57; *p* < 0.001), and rCBF (r = 0.24; *p* = 0.03), but not with rCBV (r = 0.12; *p* = 0.25). Similar results were obtained after stratification by the collateral status and DS severity **(Supplemental Table 4)**.

**Supplemental Tables**

**Supplemental Table 1 Detailed 4D-CTA protocol**

|  | **4D-CTA** |
| --- | --- |
| Scanner | Second-generation dual-source CT |
| Scan time | 37.5 s, (initially one scan every 1.5 s then every 3 s for 21 individual data sets) |
| Tube voltage | 80 kVp |
| Effective tube current | 100 mAs |
| Rotation time | 0.28 s |
| Slice collimation | 0.6 mm |
| Slice thick | 0.75 mm for dynamic CTA imaging and 5 mm for perfusion analysis |
| Injection rate | 5.0 ml/s |
| Contrast agent volume | 30 ml / 20 ml saline |
| Delay time | 5 s |

4D-CTA = four-dimensional computed tomographic angiography.

**Supplemental Table 2 Baseline characteristics of patients stratified by cerebral perfusion**

| **Variables** | **Normal perfusion**  **(N = 46)** | **Hypoperfusion**  **(N = 78)** | ***p*-value** |
| --- | --- | --- | --- |
| Age (y) | 57.0 ± 6.7 | 58.3 ± 7.2 | 0.30 |
| Male sex, n(%) | 34 (23.3%) | 62 (50%) | 0.47 |
| Current smoker, n (%) | 21 (16.9%) | 47 (37.9%) | 0.11 |
| Hypertension, n (%) | 18 (14.5%) | 57 (46.0%) | < 0.001 |
| Diabetes mellitus, n (%) | 8 (7.7%) | 13 (10.5%) | 0.91 |
| Systolic blood pressure (mmHg) | 147 (133 - 158) | 142 (131 - 150) | 0.35 |
| Diastolic blood pressure (mmHg) | 78 (67 - 86) | 81 (69 - 90) | 0.24 |
| Fasting blood glucose (mmol/L) | 6.0 ± 1.7 | 5.6 ± 1.5 | 0.12 |
| Total cholesterol (mmol/L) | 3.7 ± 1.2 | 3.6 ± 1.0 | 0.73 |
| HDL (mmol/L) | 1.1 ± 0.3 | 1.1 ± 0.4 | 0.90 |
| LDL (mmol/L) | 2.1 ± 0.7 | 2.1 ± 1.0 | 0.87 |
| Triglycerides (mmol/L) | 1.5 ± 0.8 | 1.6 ± 1.0 | 0.61 |
| Admitting diagnosis, n(%) |  |  | 0.48 |
| Transient ischemic attack | 12 (9.7%) | 25 (20.1%) |  |
| Ischemic stroke | 34 (27.4%) | 53 (42.7%) |  |
| Time from onset to CT exam (d) | 21 (14 - 33) | 17 (11 - 26) | 0.17 |
| Treatment, n (%) |  |  | 0.28 |
| OMT | 37 (80.4%) | 56 (71.8%) |  |
| EVT | 9 (19.6%) | 22 (28.2%) |  |
| Recurrent ischemic event, n (%) | 5 (6.5%) | 14 (20.5%) | 0.29 |
| Stenosis severity, n (%) |  |  | 0.05 |
| Severe | 29 (23.4%) | 63 (50.8%) |  |
| Moderate | 12 (9.7%) | 13 (10.5%) |  |
| Mild | 5 (4.0%) | 2 (1.6%) |  |
| Good collateral status, n (%) | 32 (69.6%) | 39 (50.0%) | 0.03 |
| CT-FFR | 0.84 ± 0.06 | 0.73 ± 0.09 | < 0.001 |
| CT-FFR ≤ 0.80, n (%) | 12 (26.1%) | 62 (78.5%) | < 0.001 |

HDL = high density lipoprotein; LDL = low density lipoprotein; OMT = optimal medical treatment; EVT = endovascular treatment; CT-FFR = CT-derived fractional flow reserve.

**Supplemental Table 3 Univariable and multivariable logistic regression models for predicting cerebral hypoperfusion on CT perfusion**

| **Variables** | **Univariable model** | | **Multivariable model** | |
| --- | --- | --- | --- | --- |
|  | **OR (95%CI)** | ***p*-value** | **OR (95%CI)** | ***p*-value** |
| Age (y) | 0.97 (0.91 - 1.06) | 0.59 |  |  |
| Gender (M vs F) | 0.52 (0.32 - 1.58) | 0.32 |  |  |
| Current smoker (Y vs N) | 1.98 (0.72 - 13.60) | 0.29 |  |  |
| Hypertension (Y vs N) | 5.14 (1.23 - 45.12) | 0.04 | 4.87 (1.52 - 27.92) | 0.03 |
| Diabetes mellitus  (Y vs N) | 0.82 (0.21 - 3.65) | 0.41 |  |  |
| Stenosis severity  (severe vs non-severe) | 1.37 (0.86 – 3.32) | 0.06 |  |  |
| Collateral status  (Good vs Poor) | 0.47 (0.29 - 1.07) | 0.04 | 0.40 (0.31 – 0.95) | 0.04 |
| CT-FFR  (≤ 0.80 vs > 0.80) | 6.37 (1.15 - 48.63) | 0.03 | 5.92 (1.42 – 41.12) | 0.02 |

M = male; F = female; Y = yes; N = No; CT-FFR = CT-derived fractional flow reserve.

**Supplemental Table 4 Correlations between CT-FFR and perfusion parameters**

| **CTP parameter** | **ALL** | | **Collateral status** | | | | **Stenosis severity** | | | |
| --- | --- | --- | --- | --- | --- | --- | --- | --- | --- | --- |
|  |  |  | **Poor**  **(n = 53, 42.7%)** | | **Good**  **(n = 71, 57.3%)** | | **Severe**  **(n = 92, 74.2%)** | | **Non-severe**  **(n = 32, 25.8%)** | |
|  | r | *p*-value | r | *p*-value | r | *p*-value | r | *p*-value | r | *p*-value |
| rTTP | -0.49 | < 0.001 | -0.45 | < 0.001 | -0.31 | 0.04 | -0.48 | 0.02 | -0.42 | 0.01 |
| rMTT | -0.57 | < 0.001 | -0.52 | < 0.001 | -0.37 | 0.02 | -0.55 | < 0.001 | -0.48 | < 0.001 |
| rCBF | 0.24 | 0.36 | 0.18 | 0.55 | 0.09 | 0.82 | 0.16 | 0.51 | 0.11 | 0.73 |
| rCBV | 0.12 | 0.25 | 0.07 | 0.68 | 0.03 | 0.97 | 0.05 | 0.46 | 0.10 | 0.82 |

rTTP = relative time to peak; rMTT = relative mean transit time; rCBF = relative cerebral blood flow (CBF); rCBV = relative cerebral blood volume.

**Supplemental Table 5 Diagnostic performances of different dichotomized CT-FFR**

| **CT-FFR** | **Statistical results (95% CI)** | | | | | |
| --- | --- | --- | --- | --- | --- | --- |
|  | **Sens. (%)** | **Spec. (%)** | **PPV (%)** | **NPV (%)** | **Acc. (%)** | **AUC** |
| ≤ 0.50 | 6.4 (2.2 - 14.3) | 97.8 (88.6 - 99.9) | 80.0 (29.9 - 99.0) | 37.8 (29.2 - 47.2) | 39.5 (31.4 - 48.3) | NA |
| ≤ 0.55 | 12.8 (6.3 - 22.4) | 97.8 (88.6 - 99.9) | 90.9 (57.1 - 99.5) | 39.8 (30.9 - 49.5) | 44.4 (35.9 - 53.1) | 0.55 (0.46 - 0.64) |
| ≤ 0.60 | 20.5 (12.3 - 31.2) | 97.8 (88.6 - 99.9) | 94.1 (69.2 - 99.7) | 42.1 (32.7 - 52.0) | 50.0 (41.3 - 58.8) | 0.59 (0.50 - 0.68) |
| ≤ 0.65 | 28.2 (18.6 - 39.6) | 97.8 (88.6 - 99.9) | 95.7 (76.0 - 99.8) | 44.6 (34.8 - 54.8) | 54.8 (46.1 - 63.3) | 0.62 (0.53 - 0.71) |
| ≤ 0.70 | 43.6 (32.4 - 55.4) | 93.5 (82.2 - 98.6) | 91.4 (75.8 - 97.8) | 48.3 (37.7 - 59.1) | 61.3 (52.5 - 69.4) | 0.67 (0.58 - 0.75) |
| ≤ 0.75 | 64.1 (52.4 - 74.8) | 87.0 (73.7 - 95.2) | 89.3 (77.5 - 95.6) | 58.8 (46.2 - 70.4) | 72.6 (64.1 - 79.7) | 0.75 (0.67 - 0.83) |
| ≤ 0.80 | 79.5 (68.9 - 87.8) | 73.9 (59.0 - 85.7) | 83.8 (73.0 - 91.0) | 68.0 (53.2 - 80.1) | 77.4 (69.3 - 83.9) | 0.77 (0.68 - 0.84) |
| ≤ 0.85 | 91.0 (82.5 - 96.3) | 41.3 (27.0 - 56.8) | 72.5 (62.4 - 80.8) | 73.1 (52.0 - 87.7) | 72.6 (64.1 - 79.7) | 0.66 (0.57 - 0.74) |
| ≤ 0.90 | 97.4 (91.1 - 99.8) | 15.2 (6.4 - 29.0) | 66.1 (56.6 - 74.5) | 77.8 (40.2 - 96.1) | 66.9 (58.3 - 74.6) | 0.56 (0.47 - 0.65) |

CT-FFR = CT-derived fractional flow reserve; CI = confidence interval; Sens. = sensitivity; Spec. = specificity; PPV = positive predictive value; NPV = negative predictive value; ACC. = accuracy; AUC = area under the curve; NA = nonavailable.

**Supplemental Figure Legends**

**Supplemental Figure 1** **Illustration of cerebral perfusion assessment.** Six circular ROIs were symmetrically drawn in the bilateral MCA territories at the centrum semiovale, corona radiata, and basal ganglia level.

**Supplemental Figure 2 Computational fluid dynamics (CFD) modeling procedures. (A)** Cerebral 4D-CTA source images. **(B)** Three-dimensional vessel geometry reconstruction. **(C)** Computation of mesh to generate millions of vertices and elements. **(D)** Boundary conditions, wall conditions and blood properties are defined in the model for simulation cerebral blood flow. **(E)** An established CFD model used for CT-FFR visualization.

**Supplemental Figure 3** **Relationship between recurrent ischemic event and continuum value of CT-FFR and DS.** The incidence and relative hazard of 0.05 strata CT-FFR and 10% strata DS are shown in **(A)** and **(B)**, respectively.

CT-FFR = CT-derived fractional flow reserve, DS = diameter stenosis.

**Supplemental Figure 4** **Association of CT-FFR, diameter stenosis and recurrent ischemia events one year after index stroke. (A)** The match and mismatch number between CT-FFR and diameter stenosis (DS). The data within parentheses are percentage. **(B)** The rate of recurrent ischemia event among positive match group, mismatch group, and negative match group.

CT-FFR = CT-derived fractional flow reserve, DS = diameter stenosis.

**Supplemental Figure 5 The clinical management strategies and reclassifications of CTA and CT-FFR compared to actual clinical management.**

CT-FFR = CT-derived fractional flow reserve, DS = diameter stenosis, EVT = endovascular treatment, OMT = optimal medical treatment.
